# Promoting generic drug usage in Japan: correlation between generic drug usage and monthly personal income

**DOI:** 10.1186/s40545-023-00532-5

**Published:** 2023-02-22

**Authors:** Takaaki Suzuki, Mari Iwata, Mika Maezawa, Misaki Inoue, Riko Satake, Wataru Wakabayashi, Keita Oura, Hideyuki Tanaka, Sakiko Hirofuji, Koumi Miyasaka, Fumiya Goto, Satoshi Nakao, Mayuko Masuta, Kazuhiro Iguchi, Mitsuhiro Nakamura

**Affiliations:** 1 Laboratory of Drug Informatics, Gifu Pharmaceutical University, 1-25-4, Daigaku Nishi, Gifu, 501-1196 Japan; 2 Yanaizu-Branch, Kifune Pharmacy, 2-23-2, Yanaizucho Hasuike, Gifu, 501-6103 Japan; 3 Chubu Yakuhin Co. Ltd., 4-29 Takane-cho, Tajimishi, Gifu 507-0078 Japan; 4 Division of Pharmacy, Kyushu University Hospital, 3-1-1 Maidashi, Higashi-ku, Fukuoka, 812-8582 Japan; 5 Division of Pharmacy, Kyoto City Hospital, 1-2, Mibu Higashitakadacho, Nakagyo-ku, Kyoto, 604-8845 Japan; 6 Laboratory of Community Pharmacy, Gifu Pharmaceutical University, 1-25-4, Daigaku Nishi, Gifu, 501-1196 Japan

**Keywords:** Generic drug, Monthly personal income, NDB Open Data Japan

## Abstract

**Background:**

To reduce pharmacy-related medical expenses, it is necessary to cut drug costs, potentially by increasing generic drug usage. This study analyzes the correlation between generic drug usage and monthly personal income by examining prescriptions for individual drugs.

**Methods:**

We conducted a cross-sectional study based on the data set from the National Database of Health Insurance Claims and Specific Health Checkups of Japan Open Data Japan and the Basic Survey on Wage Structure. We calculated the correlation coefficient between the usage rate of generic drugs in each prefecture of Japan and monthly personal incomes. We then analyzed the correlation coefficients based on the therapeutic categories of medicinal drugs; the contingency table was visualized as a mosaic plot. To compare the proportions between multiple categories, the chi-squared test was applied as a statistical significance test that was used in the analysis of *n* × *m* contingency tables. We worked with the null hypothesis that there were no differences between classes in the population.

**Results:**

Regarding the correlation coefficient between the usage rate of generic drugs and monthly personal incomes, the proportion of negative correlation coefficients for outpatient out-of-hospital and outpatient in-hospital prescriptions was over 70%, while that for inpatient prescriptions was 46.9%. The proportion of medicinal drugs exhibiting a negative correlation between the rates of generic drug usage and monthly personal incomes for outpatient out-of-hospital prescriptions and outpatient in-hospital prescriptions was higher than that of inpatient prescriptions. The proportion of statistically correlated medicinal drugs among inpatient prescriptions was lower than that among outpatient out-of-hospital and outpatient in-hospital prescriptions. The proportions of significant negative correlations for outpatient out-of-hospital, outpatient in-hospital, and inpatient prescriptions were 30.6%, 22.7%, and 3.5%, respectively. It was also observed that the rate of generic prescription usage for outpatient out-of-hospital and in-hospital prescriptions increased as monthly personal incomes decreased. In outpatients, the therapeutic categories with strong negative correlations were vasodilators and hyperlipidemia drugs.

**Conclusions:**

Our results may help to increase the usage rate of generic drugs in different prefectures by providing useful information for promoting them throughout Japan.

**Supplementary Information:**

The online version contains supplementary material available at 10.1186/s40545-023-00532-5.

## Background

The Japanese health insurance system guarantees public medical insurance to all citizens. Citizens can freely choose medical institutions. They can also receive advanced medical care at low costs. In principle, the share of medical expenses for Japanese citizens is 10% for those aged 75 and over, 20% for those aged 70 to 74, 30% for those aged 6 to 69, and 20% for those under 5 [1]. Rising medical costs have become a concern in Japan. Medical costs in fiscal year 2019 were 43.6 trillion yen [2], which represents an increase of 2.1 trillion yen from the 41.5 trillion yen spent in fiscal year 2015. By fiscal year 2019, approximately 16,000 items [3] were listed on the official bulletin as medical drugs to be used for medical treatment and were covered by insurance at medical institutions. Medical costs related to pharmacies were 7.7 trillion yen, of which 5.7 trillion yen was attributed to the cost of drugs [4]. In 2020, the usage rate of generic drugs in the United States reached 95%, but remained 70% in Japan [5, 6]. Increasing the usage rate of generic drugs can contribute to reducing drug costs in Japan. To reduce medical costs, the “Basic Policy on Economic and Fiscal Management and Reform 2021” [7] stipulates that the rate of generic drug usage should reach more than 80% by fiscal year 2023 in each prefecture. In Japan, dispensing generic drugs and generic substitution is governed by the Rules for Health Insurance-covered Medical Institutions and Physicians and Rules for Health Insurance-covered Dispensing Pharmacies and Pharmacists [8]. In Japan, nonproprietary name prescriptions are recommended to promote the use of generic drugs [9]. It is a system in which patients can choose between brand-name drugs and generic drugs at pharmacies. A pharmacist can dispense a generic drug unless the doctor has indicated a brand-name drug in the prescription [10].

For those aged 75 and over, those belonging to high-income groups who pay 30% of their costs under Japan’s public universal insurance system are more likely to choose brand-name drugs than those in general-income groups who pay 10% of their costs [8]. In a systematic review and meta-analysis, patients with lower income (i.e., < 200% federal poverty level) were more likely to use generic drugs than those with a higher income (≥ 200% FPL; pooled OR = 1.32, 95% CI  1.15–1.52) [11]. In the United States, it has been reported that generic drug discount programs are an option to provide affordable prescription medication to low-income individuals [12]. Large hospitals use a higher percentage of generic drugs as compared to smaller medical institutions, such as clinics [13]. Yokoi and Tashiro identified a positive correlation between the prescription rate of generic drugs and the extent of separation between a medical practice and the sites of drug dispensation [14]. There are regional differences in the use of generic drugs as well [15, 16]. In addition, although the government incentivizes the use of generic drugs, antihypertensive drugs are not effective in relatively high-income areas [15]. As prescriptions comprise outpatient out-of-hospital, outpatient in-hospital, and inpatient prescriptions, it is conceivable that the correlation between the usage rate of generic drugs and monthly personal income varies according to the difference in prescriptions. However, the correlation between the usage rate of generic drugs, sorted by prescription and monthly personal income, has not been previously investigated.

There are few research reports on the overall usage of generic drugs based on comprehensive drug prescription data in Japan. To the best of our knowledge, correlations between the usage rate of generic drugs by prescription type and monthly personal income have not been investigated for each individual drug using the National Database of Health Insurance Claims and Specific Health Checkups of Japan (NDB) Open Data Japan (NODJ). Therefore, we analyzed this correlation for each individual drug using the NODJ and Basic Survey on Wage Structure (BSWS), and then evaluated the therapeutic category that demonstrates the correlation.

## Methods

### National Database of Health Insurance Claims and Specific Health Checkups of Japan Open Data Japan (NODJ)

In 2009, based on the Act on Assurance of Medical Care for Elderly People, the Ministry of Health, Labour and Welfare (MHLW) began operating the NDB. This database accumulates monthly data on health insurance claims, and annual data on specific health checkups, thus making it one of the most exhaustive national healthcare databases worldwide. Its insurance-related data are also useful in developing government policies for national healthcare insurance systems and for academic research. NDB reflects healthcare trends in Japan, because the national healthcare system covers most medical care in the country [17]. Compared to the data obtained through sampling surveys, the NDB consists of a more comprehensive data set of individuals who have received specific health checkups in Japan. On account of its national coverage, it is also suitable for understanding the healthcare conditions in each prefecture [18].

**Fig. 1:**
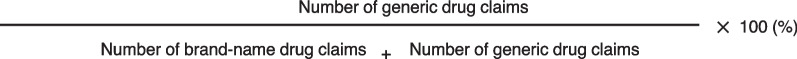
Formula for calculating the usage rate of generic drugs

The MHLW recently released the “NODJ.” This database provides a variety of NDB summaries that are freely available to the public. As the NODJ contains open data, analyses entail less effort, cost, and ethical consideration, and can be conducted quickly. We obtained data from the MHLW website for the fourth NODJ in 2017 [19]. The NODJ contains data items, such as medical and dental fee schedules, dental injuries, drug data, specified insurance medical materials, specified medical examination test items, and a specified medical examination questionnaire. The medication usage records in the fourth NODJ were restricted to the top 100 medication codes for each therapeutic category. The NODJ contains information on prescription drug usage, including outpatient out-of-hospital prescriptions, outpatient in-hospital prescriptions, and inpatient prescriptions for internal use, external use, and injection, based on the standard unit of drug price listings. We evaluated the effects of drugs based on the therapeutic categories set by the Standard Commodity Classification Number of Japan [20]. We used medications stratified by 47 Japanese prefectures, therapeutic categories, and drug prices from data tables 000711952.xlsx, 000711957.xlsx, and 000711961.xlsx [19]. We then calculated the usage rates of generic drugs stratified by outpatient out-of-hospital prescriptions, outpatient in-hospital prescriptions, and inpatient prescriptions. Medicinal drugs were classified into brand-name and generic drugs by their commercial names, and the usage rate of generic drugs was calculated according to the method prescribed by Ono et al. (Fig. 1) [21].

### Basic Survey on Wage Structure (BSWS)

In Japan, various large-scale medical information databases have been developed for clinical and epidemiological research purposes [22, 23]. Since 1948, the MHLW has annually conducted the “BSWS” [24, 25], based on the “Statistics Law.” The BSWS was conducted in 49,541 offices across the country in fiscal year 2017, aiming to present a clear picture of the employee/wage structure in major industries [25], defined as wage distribution stratified according to the types of employment and work, occupation, sex, age, school career, length of service, and occupational career. BSWS data are accessible to the public, and the data from 2017 are available from the MHLW and e-Stat websites (www.e-stat.go.jp) [26]. In this study, monthly personal income by prefecture was considered.

### Data analysis

We conducted a cross-sectional study based on the data set from the NODJ and BSWS. We calculated the correlation coefficient between the usage rate of generic drugs in each prefecture and monthly personal incomes (Additional file 1: Table S1). We then analyzed correlation coefficients based on the therapeutic categories of medicinal drugs; the contingency table was visualized as a mosaic plot. To compare the proportions between multiple categories, the chi-squared test was applied as a statistical significance test used in the analysis of *n* × *m* contingency tables. We worked with the null hypothesis that there were no differences between classes in the population. A *p* value of less than 0.05 (typically ≤ 0.05) was considered statistically significant.

The data analysis was performed using the JMP Pro 11 software (SAS Institute, Cary, NC, United States).

## Results

### Numbers of drugs by prescription category

**Fig. 2:**
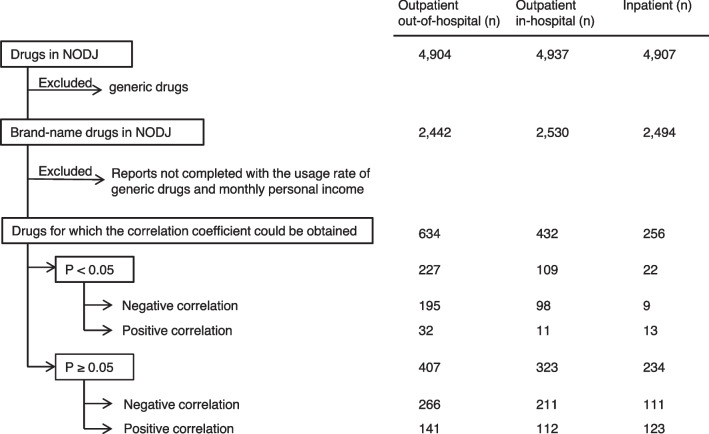
Flowchart of the analysis results for each prescription in the outpatient out-of-hospital, outpatient in-hospital, and inpatient prescription categories

The numbers of medicinal drugs for outpatient out-of-hospital, outpatient in-hospital, and inpatient prescriptions were 4904, 4937, and 4907, respectively (Fig. 2). The numbers of brand-name drugs that exhibited a significant negative correlation between the usage rate of generic drugs and monthly personal incomes for outpatient out-of-hospital, outpatient in-hospital, and inpatient prescriptions were 195, 98, and 9, respectively (Fig. 2).

### Correlation coefficients and prescription classifications (mosaic plot)

**Fig. 3:**
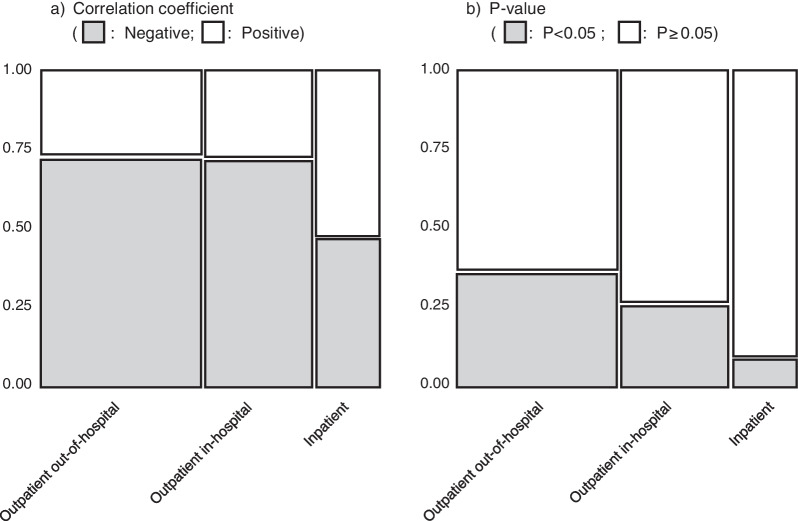
Mosaic plot of the correlation coefficients between the usage rate of generic drugs and monthly personal incomes

Using a mosaic plot, we summarized the proportion of positive/negative ratios of correlation coefficients and prescription classifications (outpatient out-of-hospital, outpatient in-hospital, and inpatient) (Fig. 3). The following significantly different categorical features according to prescription classification were detected using the chi-squared test. Regarding the correlation coefficient between the usage rate of generic drugs and monthly personal incomes, the proportion of negative correlation coefficients for outpatient out-of-hospital and outpatient in-hospital prescriptions was over 70%, while that for inpatient prescriptions was 46.9% (Fig. 3a). The proportion of inpatient prescriptions was significantly lower than that of outpatient out-of-hospital and outpatient in-hospital prescriptions (Fig. 3b).

### Proportions of significant negative correlations by prescription category

**Fig. 4:**
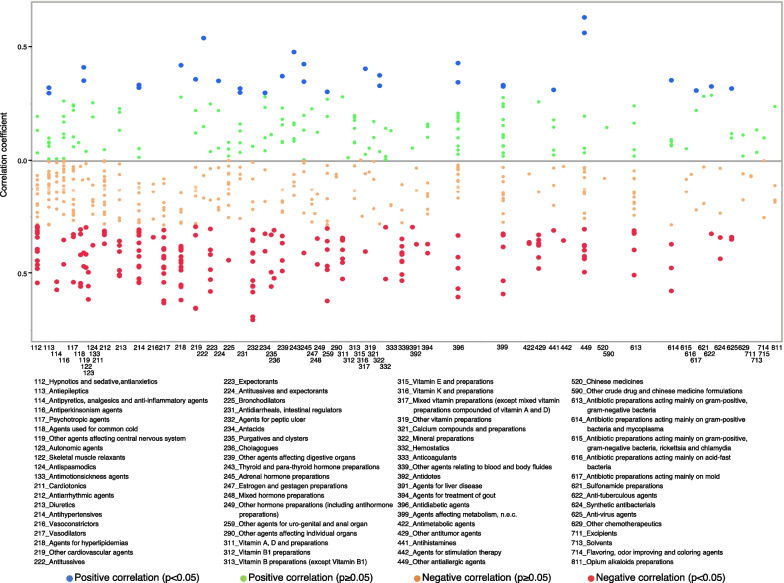
Correlation coefficients between the usage rate of generic drugs and monthly personal incomes for outpatient out-of-hospital prescriptions

The proportions of significant negative correlations for outpatient out-of-hospital, outpatient in-hospital, and inpatient prescriptions were 30.6%, 22.7%, and 3.5%, respectively. For the therapeutic categories with a negative correlation for outpatient out-of-hospital prescriptions, the numbers of antihypertensives (code: 214), vasodilators (code: 217), agents for peptic ulcer (code: 232), and agents for hyperlipidemia (code: 218) were 16, 13, 12, and 12, respectively (Table 1 and Fig. 4). The correlation coefficients for Marzulene® S-blended granules (l-glutamine, agents for peptic ulcer, code: 232), Gaster® D tablets 20 mg (Famotidine, agents for peptic ulcer, code: 232), and Cerocral® tablets 20 mg (Ifenprodil tartrate, other cardiovascular agents, code: 219) were - 0.7058 (*p* value < 0.0001), - 0.6922 (*p* value < 0.0001), and - 0.656 (*p* value < 0.0001), respectively (Additional file 2: Table S2).

**Table 1:** Numbers of brand-name drugs for which we can calculate the correlation between the usage rate of generic drugs and monthly personal incomes based on NODJ with *p* values less than 0.05

Code	Therapeutic categories of standard commodity classification number	Outpatient out-of-hospital				Outpatient in-hospital				Inpatient			
		Total (all)	Total (*p* < 0.05)	Negative correlation	Positive correlation	Total (all)	Total (*p* < 0.05)	Negative correlation	Positive correlation	Total (all)	Total (*p* < 0.05)	Negative correlation	Positive correlation
112	Hypnotics and sedative, antianxietics	27	11	11	0	19	8	8	0	16	1	1	0
113	Antiepileptics	24	2	0	2	16	2	1	1	13	0	0	0
114	Antipyretics, analgesics and anti-inflammatory agents	10	2	2	0	8	1	1	0	2	0	0	0
116	Antiperkinsonism agents	20	2	2	0	7	1	1	0	8	2	1	1
117	Psychotropic agents	21	3	3	0	12	2	2	0	19	0	0	0
118	Agents used for common cold	1	0	0	0	1	0	0	0	1	0	0	0
119	Other agents affecting central nervous system	7	6	6	0	4	4	4	0	2	0	0	0
121	Local anesthetics	0	0	0	0	1	0	0	0	1	0	0	0
122	Skeletal muscle relaxants	1	0	0	0	0	0	0	0	0	0	0	0
123	Autonomic agents	4	4	2	2	3	0	0	0	1	0	0	0
124	Antispasmodics	5	3	3	0	4	2	2	0	4	1	1	0
133	Antimotionsickness agents	4	3	3	0	3	3	3	0	2	0	0	0
211	Cardiotonics	8	1	1	0	6	2	1	1	1	0	0	0
212	Antiarrhythmic agents	19	4	4	0	15	7	7	0	7	0	0	0
213	Diuretics	16	6	6	0	10	2	2	0	8	1	1	0
214	Antihypertensives	29	18	16	2	21	3	3	0	9	2	0	2
216	Vasoconstrictors	4	1	1	0	1	1	1	0	1	0	0	0
217	Vasodilators	20	13	13	0	16	11	11	0	9	0	0	0
218	Agents for hyperlipidemias	19	13	12	1	15	10	9	1	5	0	0	0
219	Other cardiovascular agents	14	6	5	1	8	3	3	0	7	1	1	0
222	Antitussives	4	1	0	1	4	0	0	0	2	1	0	1
223	Expectorants	11	7	7	0	7	2	2	0	6	0	0	0
224	Antitussives and expectorants	7	1	0	1	3	0	0	0	0	0	0	0
225	Bronchodilators	15	1	1	0	9	2	2	0	2	1	0	1
231	Antidiarrheals, intestinal regulators	10	2	0	2	8	1	1	0	5	0	0	0
232	Agents for peptic ulcer	17	12	12	0	15	8	8	0	11	1	1	0
234	Antacids	9	3	2	1	7	3	1	2	3	1	1	0
235	Purgatives and clysters	4	3	3	0	4	0	0	0	3	0	0	0
236	Cholagogues	2	2	2	0	2	0	0	0	2	0	0	0
239	Other agents affecting digestive organs	15	5	4	1	11	3	2	1	6	0	0	0
243	Thyroid and para-thyroid hormone preparations	9	1	0	1	5	0	0	0	3	2	0	2
245	Adrenal hormone preparations	11	3	1	2	9	1	0	1	7	2	1	1
247	Estrogen and gestagen preparations	5	0	0	0	2	1	1	0	0	0	0	0
248	Mixed hormone preparations	1	0	0	0	1	1	1	0	0	0	0	0
249	Other hormone preparations (including antihormone preparations)	4	2	2	0	3	2	1	1	1	0	0	0
253	Oxytocics	0	0	0	0	0	0	0	0	1	0	0	0
259	Other agents for uro-genital and anal organ	15	7	6	1	13	2	2	0	3	0	0	0
290	Other agents affecting individual organs	2	0	0	0	0	0	0	0	0	0	0	0
311	Vitamin A, D and preparations	8	7	7	0	5	2	2	0	0	0	0	0
312	Vitamin B1 preparations	1	0	0	0	1	0	0	0	1	0	0	0
313	Vitamin B preparations (except Vitamin B1)	9	0	0	0	8	0	0	0	3	0	0	0
315	Vitamin E and preparations	1	0	0	0	1	0	0	0	0	0	0	0
316	Vitamin K and preparations	1	0	0	0	1	0	0	0	1	0	0	0
317	Mixed vitamin preparations (except mixed vitamin preparations compounded of vitamin A and D)	4	2	1	1	2	0	0	0	2	0	0	0
319	Other vitamin preparations	2	0	0	0	0	0	0	0	0	0	0	0
321	Calcium compounds and preparations	5	0	0	0	1	0	0	0	1	0	0	0
322	Mineral preparations	4	2	0	2	3	0	0	0	4	1	0	1
323	Saccharide preparations	0	0	0	0	0	0	0	0	1	0	0	0
332	Hemostatics	5	2	2	0	3	0	0	0	2	0	0	0
333	Anticoagulants	3	0	0	0	3	0	0	0	2	0	0	0
339	Other agents relating to blood and body fluides	13	10	10	0	13	5	5	0	9	0	0	0
391	Agents for liver disease	2	1	1	0	3	0	0	0	1	0	0	0
392	Antidotes	3	1	1	0	1	1	1	0	1	0	0	0
394	Agents for treatment of gout	11	3	3	0	5	1	1	0	2	0	0	0
396	Antidiabetic agents	27	7	5	2	18	2	2	0	7	1	0	1
399	Agents affecting metabolism, n.e.c.	29	7	5	2	15	4	3	1	11	1	0	1
422	Antimetabolic agents	3	2	2	0	2	0	0	0	2	0	0	0
429	Other antitumor agents	8	6	6	0	1	0	0	0	0	0	0	0
441	Antihistamines	9	2	1	1	5	0	0	0	1	0	0	0
442	Agents for stimulation therapy	2	1	1	0	1	0	0	0	0	0	0	0
449	Other antiallergic agents	21	9	7	2	16	0	0	0	5	0	0	0
520	Chinese medicines	1	0	0	0	11	0	0	0	8	1	1	0
590	Other crude drug and chinese medicine formulations	1	0	0	0	0	0	0	0	0	0	0	0
613	Antibiotic preparations acting mainly on gram-positive, gram-negative bacteria	21	5	5	0	14	4	3	1	5	1	0	1
614	Antibiotic preparations acting mainly on gram-positive bacteria and mycoplasma	9	4	3	1	5	1	1	0	0	0	0	0
615	Antibiotic preparations acting mainly on gram-positive, gram-negative bacteria, rickettsia and chlamydia	3	0	0	0	2	0	0	0	2	0	0	0
616	Antibiotic preparations acting mainly on acid-fast bacteria	1	0	0	0	0	0	0	0	0	0	0	0
617	Antibiotic preparations acting mainly on mold	3	1	0	1	0	0	0	0	2	1	0	1
621	Sulfonamide preparations	3	0	0	0	2	0	0	0	1	0	0	0
622	Anti-tuberculous agents	3	2	1	1	1	0	0	0	1	0	0	0
624	Synthetic antibacterials	4	2	2	0	5	0	0	0	1	0	0	0
625	Anti-virus agents	5	3	2	1	2	0	0	0	1	0	0	0
629	Other chemotherapeutics	4	0	0	0	2	0	0	0	1	0	0	0
711	Excipients	2	0	0	0	1	0	0	0	0	0	0	0
713	Solvents	2	0	0	0	1	1	0	1	1	0	0	0
714	Flavoring, odor improving and coloring agents	3	0	0	0	1	0	0	0	1	0	0	0
721	X-ray contrast agents	0	0	0	0	1	0	0	0	1	0	0	0
729	Other diagnostic agents (except extracorporeal diagnostic medicines)	0	0	0	0	1	0	0	0	0	0	0	0
799	Agents for not mainly purpose of therapeutic, n.e.c.	0	0	0	0	1	0	0	0	1	0	0	0
811	Opium alkaloids preparations	4	0	0	0	1	0	0	0	3	0	0	0

**Fig. 5:**
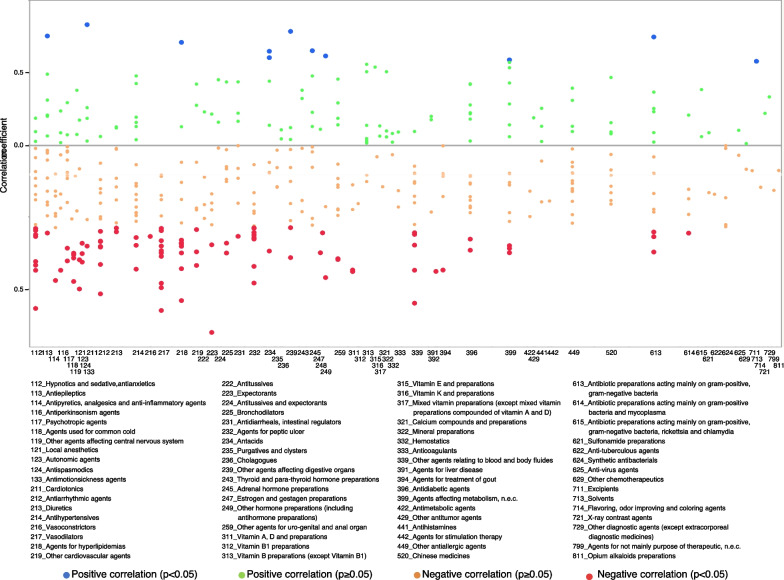
Correlation coefficients between the usage rate of generic drugs and monthly personal incomes for outpatient in-hospital prescriptions

Regarding the therapeutic categories with negative correlations for outpatient in-hospital prescriptions, vasodilators (code: 217), agents for hyperlipidemias (code: 218), agents for peptic ulcer (code: 232), and hypnotics and sedatives, antianxietics (code: 112) accounted for 11, 9, 8, and 8 items, respectively (Table 1 and Fig. 5). The correlation coefficients of Mucosolvan® Tablets 15 mg (Ambroxol hydrochloride, expectorants, code: 223), Amlodin® Tablets 5 mg (Amlodipine besilate, vasodilators, code: 217), and Lendormin® Tablets 0.25 mg (Brotizolam, hypnotics and sedatives, antianxietics, code: 112) were - 0.6504 (*p* value < 0.0001), - 0.5744 (*p* value < 0.0001), and - 0.5675 (*p* value < 0.0001), respectively (Additional file 3: Table S3).

**Fig. 6:**
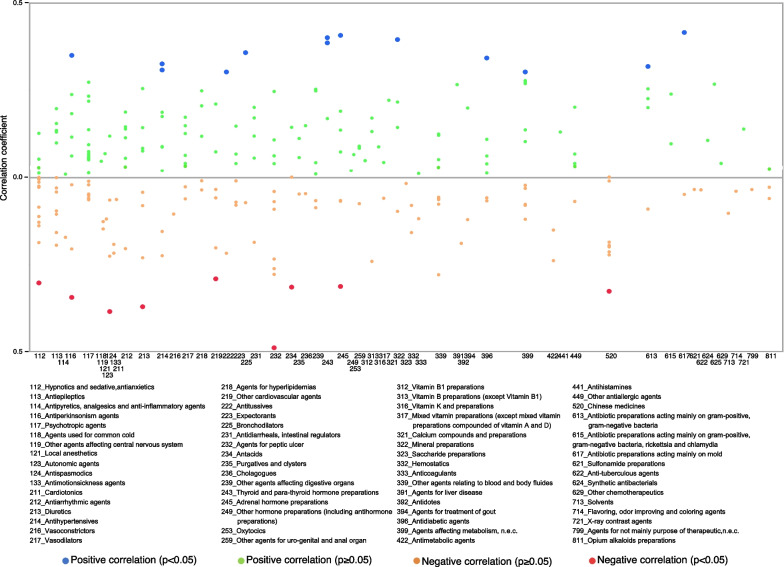
Correlation coefficients between the usage rate of generic drugs and monthly personal incomes for inpatient prescriptions

Regarding the therapeutic categories with negative correlations for inpatient prescriptions, hypnotics and sedatives, antianxietics (code: 112), antiparkinsonism agents (code: 116), antispasmodics (code: 124), diuretics (code: 213), other cardiovascular agents (code: 219), agents for peptic ulcer (code: 232), antacids (code: 234), adrenal hormone preparations (code: 245), and Chinese medicines (code: 520) accounted for 1, 1, 1, 1, 1, 1, 1, 1, and 1 items, respectively (Table 1 and Fig. 6). The correlation coefficients for Marzulene® S-blended granules (l-glutamine, agents for peptic ulcers, code: 232), Ternerin® tablets 1 mg (Tizanidine Hydrochloride, antispasmodics, code: 124), and Diart® tablets 60 mg (Azosemide, diuretics, code: 213) were - 0.4904 (*p* = 0.0006), - 0.3864 (*p* value = 0.0073), and - 0.3726 (*p* value = 0.0099), respectively (Additional file 4: Table S4).

The average drug prices (Japanese yen: mean ± standard deviation) associated with significant negative correlations between the usage rate of generic drugs and monthly personal incomes for outpatient out-of-hospital, outpatient in-hospital, and inpatient prescriptions were 91.4 ± 150.2, 71.1 ± 113.8, and 13.8 ± 9.2, respectively (Table 2).

**Table 2:** Drug prices for which we can calculate the correlation between the usage rate of generic drugs and monthly personal incomes

Item	Drug prices (all)	Drug prices (significantly correlated) (Japanese yen)		
		Total	Negative correlation	Positive correlation
*Outpatient out-of-hospital*				
Number	634	227	195	32
Mean ± SD^*^	91.1 ± 253.5	91.3 ± 160.1	91.4 ± 150.2	90.6 ± 210.4
Median (min–max)	27.5 (0.1–3476.9)	40.6 (0.7–1139.2)	43.5 (0.7–945.1)	16.7 (0.7–1139.2)
*Outpatient in-hospital*				
Number	432	109	98	11
Mean ± SD^*^	73.5 ± 212.7	66.7 ± 109.3	71.1 ± 113.8	28.1 ± 36.7
Median (min–max)	24.1 (0.1–2825.7)	33.8 (0.1–620.7)	37.2 (0.7–620.7)	9.6 (0.1–104.0)
*Inpatient*				
Number	256	22	9	13
Mean ± SD^*^	62.5 ± 136.4	75.3 ± 234.4	13.8 ± 9.2	117.8 ± 297.5
Median (min–max)	17.1 (0.1–1139.2)	12.5 (0.7–1139.2)	13.0 (0.7–31.7)	11.9 (5.6–1139.2)

^*^Standard deviation

## Discussion

In Japan's medical system, additional insurance premiums are charged according to the usage rate of generic drugs for outpatient out-of-hospital prescriptions, outpatient in-hospital prescriptions, and inpatient prescriptions [27]. However, the usage rate of generic drugs differs among the three. The self-pay rate is essentially 10% for those aged 75 and over, 20% for those aged 70 to 74, 30% for those aged 6 to 69, and 20% for those under 5 [1]. For outpatient out-of-hospital prescriptions and outpatient in-hospital prescriptions, the proportion of medicinal drugs that exhibited a negative correlation between the usage rate of generic drugs and monthly personal incomes was higher than that of inpatient prescriptions (Fig. 3a). The proportion of statistically correlated medicinal drugs for inpatient prescriptions was lower than that for outpatient out-of-hospital and outpatient in-hospital prescriptions (Fig. 3b). This result could be due to the difference in the system of additional insurance premiums for the use of generic drugs among the three groups of patients. Another possible reason is that inpatients are more likely to be aged 70 or more, and have a lower self-pay rate than outpatients [28]. To promote the use of generic drugs, it may be necessary to review the premium system for the use of generic drugs in in-hospital prescriptions.

Moreover, the rate of generic prescription usage for outpatient out-of-hospital and outpatient in-hospital prescriptions increased as monthly personal incomes decreased (Fig. 3a). This result is consistent with previous research findings that show that incentivizing the use of generic antihypertensive drugs was not effective in areas with relatively high-income levels [15]; for the elderly aged 75 and over, those in high-income groups are more likely to choose brand-name drugs than those in general-income groups [8]. These results may contribute to policies targeted at reducing medical costs. For outpatients, the therapeutic categories related to negative correlations were vasodilators and hyperlipidemia drugs. The generic usage rates of these categories of drugs prescribed nationwide for the treatment of lifestyle-related diseases negatively correlated with monthly personal incomes. In addition, we investigated the correlation coefficient for each drug, with no clear trend identified (Figs. 4, 5, 6). Federman et al. reported that the elderly with low incomes or no prescription coverage were more likely to use generic cardiovascular drugs than high-income and insured seniors [29]. A possible reason for this is that people with lower incomes need to reduce their expenditure, and this is done by reducing the cost of drugs for treating lifestyle-related diseases that need to be purchased on a regular basis.

For medicinal drugs with significant negative correlations, the median price for outpatient out-of-hospital and outpatient in-hospital prescriptions was higher than that of inpatient prescriptions (Table 2). This indicates that a high-priced, brand-name drug may be prescribed as a generic drug outside of hospitals. This result, which is related to monthly personal income by region and the usage rate of generic drugs, suggests that the usage of generic drugs reduces medical costs. It is important to consider economic measures to promote the usage of generic drugs among high-income people to further increase the usage rate of generic drugs.

The average price of brand-name drugs that are significantly positive correlated with outpatient in-hospital care was the lowest among the prescription categories. Although this is an interesting discovery, an investigation of the reasons must be reserved for future studies.

Several limitations should be considered when interpreting the NODJ results. The use of medications in the NODJ database is restricted to the top 100 medication codes in each therapeutic category, and not all the NDB medication codes were included. Therefore, the drugs studied were not representative of all the drugs in their respective therapeutic categories. The ratio of the drugs surveyed to all drugs was assumed to vary according to their category.

This study investigated the correlations at the regional prefectural level. Regional correlation studies cannot evaluate the associations between individual-level exposure and outcomes, nor can they elucidate the causal relationships and risk assessment of the usage rate of generic drugs at the individual level. It is important to consider these factors when a prefecture develops measures to increase the usage rate of generic drugs.

## Conclusion

Our results may help increase the usage rate of generic drugs in different prefectures by providing useful information for promoting their usage throughout Japan. Furthermore, our statistical analysis may serve as a valuable resource for policymakers attempting to reduce healthcare costs by encouraging the use of generic prescription drugs.

## Supplementary Information

**Additional file 1: Table S1.** Monthly personal income and the usage rate of generic drugs in prescriptions by each prefecture.

**Additional file 2: Table S2.** Correlation coefficients between the usage rate of generic drugs and monthly personal income for outpatient out-of-hospital prescriptions in each prefecture.

**Additional file 3: Table S3.** Correlation coefficients between the usage rate of generic drugs and monthly personal income for outpatient in-hospital prescriptions in each prefecture.

**Additional file 4: Table S4.** Correlation coefficients between the usage rate of generic drugs and monthly personal income for inpatient prescriptions in each prefecture.

## Data Availability

Data used in this study are available from the following links: https://www.mhlw.go.jp/stf/seisakunitsuite/bunya/0000177221_00003.html, www.e-stat.go.jp, https://www.e-stat.go.jp/stat-search/files?page=1&layout=datalist&toukei=00450091&tstat=000001011429&cycle=0&tclass1=000001098975&tclass2=000001098977&tclass3=000001098986&tclass4val=0.

## Abbreviations

MHLW: Ministry of Health, Labour, and Welfare
BSWS: Basic Survey on Wage Structure
NDB: National Database of Health Insurance Claims and Specific Health Checkups of Japan
NODJ: National Database of Health Insurance Claims and Specific Health Checkups of Japan from Open Data Japan
